# Incisional hernia repair following pancreatic surgery—open vs laparoscopic approach

**DOI:** 10.1007/s10029-023-02901-0

**Published:** 2023-10-30

**Authors:** C. M. Krueger, M. Patrzyk, J. Hipp, U. Adam, F. Köckerling, H. Riediger

**Affiliations:** 1 Department of Surgery, Centre of Robotics, Immanuel Clinic Ruedersdorf, University Clinic of Brandenburg Medical School, Brandenburg, Germany; 2 Department of Surgery, Clinic for General, Visceral, Vascular and Thoracic Surgery, Greifswald University Medical Centre, Greifswald, Germany; 3 Department of General and Visceral Surgery, Faculty of Medicine, Medical Center, University of Freiburg, Freiburg/Breisgau, Germany; 4 Department for Surgery, Vivantes-Humboldt-Hospital, Academic Teaching Hospital of Charité University Medicine, Berlin, Germany

**Keywords:** Incisional hernia, Pancreatic surgery, Laparoscopic IPOM, Sublay, Component separation, Transversus abdominis release

## Abstract

**Introduction:**

For pancreatic procedures, transverse and midline or combined approaches are used. Having an increased morbidity after pancreatic surgery, these patients have an increased risk of developing an incisional hernia. In the following, we will analyze how the results of incisional hernia surgery after pancreatic surgery are presented in the Herniamed Registry.

**Methods:**

Hospitals and surgeons from Germany, Austria and Switzerland can voluntarily enter all routinely performed hernia operations prospectively into the Herniamed Registry. All patients sign a special informed consent declaration that they agree to the documentation of their treatment in the Herniamed Registry. Perioperative complications (intraoperative complications, postoperative complications, complication-related reoperations and general complications) are recorded up to 30 days after surgery. After 1, 5, and 10 years, patients and primary care physicians are contacted and asked about any pain at rest, pain on exertion, chronic pain requiring treatment or recurrence. This retrospective analysis of prospectively collected data compares the outcomes of minimally invasive vs open techniques in incisional hernia repair after pancreatic surgery.

**Results:**

Relative to the total number of all incisional hernia patients in the Herniamed Registry, the proportion after pancreatic surgery with 1-year follow-up was 0.64% (*n* = 461) patients. 95% of previous pancreatic surgeries were open. Minimally invasive incisional hernia repair was performed in 17.1% and open repair in 82.9% of cases. 23.2% of the defects were larger than 10 cm and 32.8% were located laterally or were a combination of lateral and medial defects. Among the few differences between the collectives, a significantly higher rate of defect closure (58.1% vs 25.3%; *p* < 0.001) and drainage (72.8% vs 13.9%; *p* < 0.001) was found in the open repairs, and larger meshes were seen in the minimally invasive procedures (340.6 cm^2^ vs 259.6 cm^2^; *p* < 0.001). No difference deemed a risk factor for chronic postoperative pain was seen in the rate of preoperative pain between the open and minimally invasive procedures (Appendix Table 4) No significant differences were found in either the perioperative complications or at 1-year follow-up.

**Conclusions:**

Incisional hernias after complex pancreatic surgery can be repaired safely and with a low recurrence rate in both open and minimally invasive techniques.

## Introduction

Pancreatic surgery has experienced significant medical progress in the past decades and is now characterized by much lower mortality than in the past [1–3]. However, morbidity is still high in patients with pancreatic resection. Wound healing disorders continue to play a significant role. Thus, this patient group per se has an increased risk of developing incisional hernias. The main indications for pancreatic resection are still malignancies and chronic pancreatitis. In the last decade, cystic neoplasms have been added as incidental findings of precancerous disease in healthy patients. The majority of elective surgical pancreatic procedures are performed in conventional open surgery. Minimally invasive procedures have gained importance in recent years. There is, therefore, a heterogeneous group in terms of comorbidities and long-term prognosis. The risk of incisional hernia described in the literature is 12–18% [4–7].

Different access techniques are used in open pancreatic surgery. This variability results from the strategy, which must be aligned with the goal of the operation [8]. Often, surgery is performed via a transverse approach, which should be guided with a 2 cm distance from the costal arch [8]. In general, there is a lower risk of developing an incisional hernia after performing a transverse laparotomy [8, 9]. However, if a hernia does occur permanent correction is difficult [10]. Compared with medial incisional hernias, lateral incisional hernias are at significantly higher risk of recurrence [11]. To prevent incisional hernia, the combination of medial and transverse laparotomy should be avoided [8]. The use of mesh augmentation for closure of lateral incisions may reduce the incidence of incisional hernias [12]. The present analysis of data from the Herniamed Register is intended, on one hand, to shed light on the problems of treating transverse incisional hernias following complex upper abdominal procedures, such as pancreatic surgery, and on the other hand to compare the outcomes of open vs minimally invasive incisional hernia repair.

## Methods

The following analysis from the Herniamed Registry compares the outcomes of laparoscopic and open incisional hernia repair after pancreatic surgery. Both perioperative (intraoperative complications, postoperative complications, complication-related reoperations) and 1-year follow-up outcomes (recurrence rate, rates of pain at rest and on exertion, and chronic pain requiring treatment) have been studied.

**Fig. 1:**
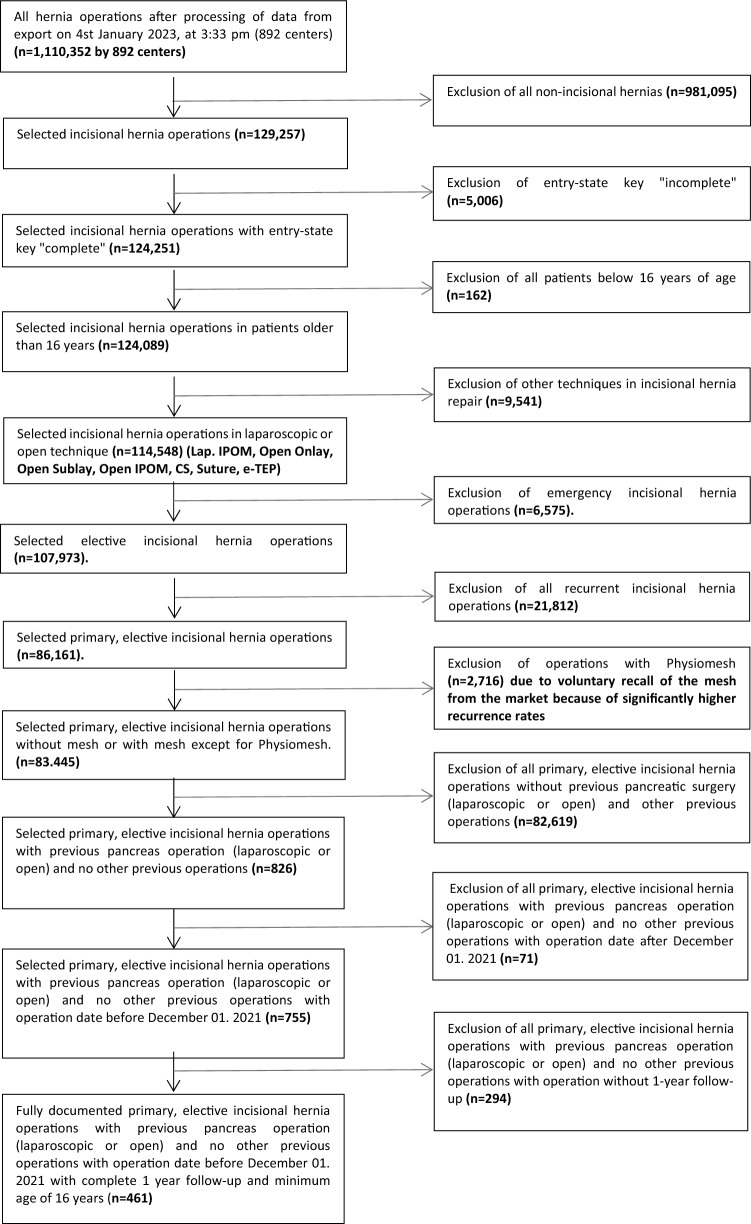
Flowchart of patient inclusion

Herniamed is an internet-based hernia registry in which hospitals and surgeons in private practice from Germany, Austria, and Switzerland can voluntarily enter their routinely performed hernia operations [10, 11]. On the cutoff date of 04 January 2023, the number of participating clinics/practices was 892 (Fig. 1). All patients signed a special informed consent form agreeing to participate in the Herniamed Registry. During the consultation for documentation in the Herniamed Registry, patients are requested to inform their treating clinic/practice about any problem after hernia surgery. If there are any problems after surgery, the patient can visit their treating clinic/practice for a clinical examination at any time. Perioperative complications are recorded up to 30 days after surgery.

After 1, 5, and 10 years, the patient and the primary care physician are sent a questionnaire asking about any pain at rest, pain on exertion, and chronic pain requiring treatment or any bulging/recurrence. Pain is graded using the visual analog scale. In addition, the patient is asked again about any perioperative complications that occurred and, if necessary, a follow-up visit is arranged [11]. If the patient or their primary care physician reports a problem at follow-up, the patient may be requested to attend the treating hospital/practice for further diagnostic measures based on clinical examination, CT, ultrasound or MRI.

The present analysis retrospectively examines the prospectively collected data of patients who underwent primary elective incisional hernia repair after previous pancreatic surgery. Open vs minimally invasive incisional hernia operations were compared.

All analyses were performed with the software SAS 9.4 (SAS Institute Inc., Cary, NC, USA) and intentionally calculated to a full significance level of 5%, i.e., they were not corrected in respect of multiple tests, and each *p* value ≤ 0.05 represents a significant result. Unadjusted analyses were performed to analyze the effect of an individual influencing factor on an outcome parameter, with the main focus on the association with the surgical procedure. For a categorical outcome variable the Chi-square test was used. For continuous outcome variables, the ANOVA (analysis of variance) was used to analyze the influence of the comparison groups.

The complete results are presented in tabular form in the appendix. Relevant partial aspects are already mentioned in the Results section of the text.

## Results

### Data of the total group

There are 129,257 patients with incisional hernias in the Herniamed Registry as of January 04, 2023. Of these, 755 patients meet the criterion of pancreatic surgery as the only previous abdominal surgery. Relative to the total number of all patients with incisional hernias in the Herniamed Registry, this is a very small group (0.58%). 1-year follow-up information after 12 months was available for 461 patients.

In 440 patients (95%), the pancreas was operated on using the classic open technique. Minimally invasive pancreatic surgery accounts for only 5% (*n* = 21) of all procedures.

Most incisional hernias were 4–10 cm in size (*n* = 240; 52.0%) and were located medially in 310 cases (67.2%). Lateral (*n* = 71; 15.4%) or combined (*n* = 80; 17.4%) incisional hernias occurred in 151 patients (32.8%). Preoperative pain was reported in 264 (57.0%) cases. In almost a quarter (*n* = 114; 24.8%) of cases, the defect size was less than 4 cm. A total of 36 (7.8%) cases used mesh-free direct suture techniques. It is not possible to say why surgeons opted for a mesh-free procedure. A defect larger than 10 cm (W3, EHS classification) was found in a roughly equal group of patients (21.5% minimally invasive vs 23.6% open; *p* = 0.926).

Surgical techniques involving retromuscular (Sublay; *n* = 234; 50.8%) or intraperitoneal mesh placement (lap. and open IPOM *n* = 160; 34.7%) were performed in 394 (85%) cases. All other techniques were only rarely used (Tab. 1). Surprisingly, the open suture technique was used in in 7.8% (*n* = 36) of cases.

**Table 1:** Perioperative data of all patients *n* = 461

		*n* (%)
Gender	Male	323 (70)
	Female	128 (30)
ASA	I	36 (7.8)
	II	260 (56.4)
	II/IV	165 (35.8)
Preoperative pain	Yes	264 (57%)
	No	158 (43%)
Size of the defect	I (< 4 cm)	114 (24.8)
	II (4–10 cm)	240 (52.0)
	III (> 10 cm)	107 (23.2)
EHS classification	Medial	310 (67.2)
	Lateral	71 (15.4)
	Combined	80 (17.4)
Access route for pancreatic surgery	Laparoscopic	21 (4.5)
	Open	440 (95.5)
Access route for hernia surgery	Minimally invasive (laparoscopic + e-TEP)	79 (17.1)
	Open	382 (82,9)
Type of hernia surgery	Open Sublay	225 (48.8)
	Open IPOM	82 (17.8)
	Laparoscopic IPOM	78 (16,9)
	Open Suture	36 (7,8)
	Open Onlay	30 (6,5)
	Component separation	9 (2.0)
	e-TEP	1 (0.2)
	Mesh implantation	421 (91.2)
	Drainage	289 (8.7)

### Previous pancreatic surgery

Only 4.5% (*n* = 21) of the previous pancreatic surgeries were performed in laparoscopic and 95.5% (*n* = 440) open technique.

### Comparison of patient collectives with endoscopic and open incisional hernia repair

Of the 461 incisional hernia operations, 79 (17.1%) were performed endoscopically and 382 (82.9%) openly.

There were only a few significant differences between endoscopic and open incisional hernia repair. For example, drainage was used significantly more often in open surgery (72.8% vs 13.9%; *p* < 0.001), as was defect closure (58.1% vs 25.3%; *p* < 0.001). The mesh used was significantly smaller in open surgery than in the minimally invasive technique (259.6 cm^2^ vs 340.6 cm^2^; *p* < 0.001). There was no significant difference in defect sizes.

No significant difference was found between the endoscopic and open incisional hernia repairs with regard to the operation techniques based on the EHS classification for incisional hernia localization: medial, lateral and combined (Table 2).

**Table 2:** Operation techniques based on the EHS classification for incisional hernia localization

Operation technique	EHS classification					
	Medial		Lateral		Combined	
	*N*	%	*N*	%	*N*	%
Laparoscopic—IPOM	57	18.39	10	14.08	11	13.75
Open—Onlay	17	5.48	8	11.27	5	6.25
Open—Sublay	144	46.45	37	52.11	44	55.00
Open—IPOM	60	19.35	8	11.27	14	17.50
Component separation	6	1.94	2	2.82	1	1.25
Open—Direct suture	25	8.06	6	8.45	5	6.25
e-TEP	1	0.32	0	0.00	0	0.00
Total	310	100.00	71	100.00	80	100.00

Up to 30 days after surgery, there was no significant difference between minimally invasive and open incisional hernia surgery after previous pancreatic surgery with regard to the perioperative complication rates (intraoperative complications, general complications, postoperative complications, complication-related reoperation) (see Tables 7, 8, 9 in the Appendix). Likewise, at 1-year follow-up, no significant differences were seen between the open and minimally invasive procedures in the rates of recurrence, pain on exertion, pain at rest, and chronic pain requiring treatment (Table 3).

**Table 3:** 12-Month follow-up for selected parameters

	Type of incisional hernia surgery		*p*
	Minimally invasive (*n* = 79)	Open (*n* = 382)	
	*n* (%)	*n* (%)	=
Recurrences	3 (3.8)	22 (5.8)	0.48
Pain on exertion	17 (21.5)	80 (20.9)	0.90
Pain at rest	8 (10.1)	44 (11.5)	0.72
Pain requiring treatment	6 (7.6)	34 (8.9)	0.70

## Discussion

In the participating countries of the Herniamed Registry (Germany, Austria, Switzerland), several thousand pancreatic procedures are performed annually. In relation to this, the number of patients with operated incisional hernias in this registry study seems very small. However, no precise figures are available on this. However, studies with larger numbers of cases often report only the occurrence of incisional hernias and their risk factors, with no information on surgical management or follow-up [5, 7, 12, 13]. It is conceivable that a relevant proportion of patients with incisional hernias after pancreatic surgery are subject to a watch and wait concept, as the hernia is not at the medical forefront.

Incisional hernia operations were performed only in 5% of patients after laparoscopic and in 95% after open prior pancreatic surgery. According to the US Nationwide Inpatient Sample database from 2000 to 2011, only 5% of all pancreatic resections were performed laparoscopically [14]. A systematic review revealed that incisional hernias occur significantly less frequently after laparoscopic abdominal surgery than after open abdominal surgery. [15]. Accordingly, efforts are being made to increase the proportion of minimally invasive pancreatic surgery cases using a surgical robot [16]. This should be able to reduce the rate of incisional hernias after pancreatic surgery.

The percentage of lateral (15.4%) and combined (17.4%) incisional hernias after pancreatic surgery is 32.8% (Table 1). Lateral and combined incisional hernias are considered complex and their treatment more difficult [8]. In addition, larger defects occur more frequently after pancreatic surgery than after other previous surgeries. For example, the percentage of incisional hernias with a defect of > 10 cm after pancreatic resection is 23.2%, while it is only 16.4% after other preoperative procedures [11]. Defects of size 4–10 cm also occur more frequently after pancreatic surgery at 52.0% compared to other abdominal surgeries at 45.9%. Based on these figures, it is clear that incisional hernia operations after previous pancreatic surgery are more difficult to treat due to the size and localization of the defects. This is then also reflected in the proportion of minimally invasive operations. While the proportion of laparoscopic incisional hernia repairs after pancreatic surgery is only 17.1%, the proportion in the total collective of primary elective incisional hernia repair is 27.8% [11].

In the comparison of the results after minimally invasive vs open incisional hernia operations after pancreatic surgery, no significant differences were found either for the perioperative results (intraoperative complications, postoperative complications, complication-related reoperation, and general complications) or at 1-year follow-up (recurrence, pain at rest, pain on exertion and chronic pain requiring treatment). However, it has to be said that in the open group the proportion of patients with defects > 10 cm, lateral and combined defect localization and ASA classification is significantly higher. Nevertheless, it seems to be possible even with minimally invasive surgery to achieve good results in the treatment of incisional hernias after such complex previous operations as surgery on the pancreas.

There are also no significant differences when compared with the results of primary elective incisional hernia surgery in other patients in the Herniamed Registry [11]. Thus, previous surgery on the pancreas with subsequent formation of an incisional hernia does not represent an argument against appropriate surgical management. The results also support a minimally invasive approach after complex surgery on the pancreas. The IPOM technique is also associated with very good results in this study. In the future, we expect an increase in component separation techniques. Except for a few findings (small medial incisional hernia), release of the transversus abdominis will always be required if adequate overlap in the extra-peritoneal plane is to be achieved. The technique is required for all lateral and combined hernias. The same is true for medial hernias with close contact to the xiphoid process. Therefore, robot-assisted surgery of incisional hernia after prior operation on the pancreas may gain increasing importance in the future [17–19].

A limiting factor for this study is that due to the complexity of the underlying disease, treatment of an existing hernia may often not be performed in a relevant number of cases. Consequently, only a small number of patients with an incisional hernia after previous pancreatic surgery are available for this registry study. For the follow-up, data are available for only about 61.1% of patients. The diversity in surgical techniques presented in this study constitutes a major confounding factor, thereby complicating the drawing of definitive conclusions. The 7.8% proportion with incisional hernia suture closure is problematic. A higher recurrence rate must be expected for this subgroup [11].

## Conclusion

The data show that surgical management of incisional hernias after pancreatic surgery is safely feasible. At 1-year follow-up, the recurrence rate is low. After pancreatic surgery, no significant differences were found either for the perioperative results (intraoperative complications, postoperative complications, complication-related reoperation, and general complications) or for the 1-year follow-up outcomes (rates of recurrence, pain at rest, pain on exertion and chronic pain requiring treatment).

## Appendix

See Appendix Tables 4, 5, 6, 7, 8, 9.

**Table 4:** Categorical parameters: patient- and procedure-related parameters, and risk factors

		Access route for hernia surgery				*p*
		Minimally invasive		Open		
		*n*	%	*n*	%	
Sex	Male	61	77.2	262	68.6	0.127
	Female	18	22.8	120	31.4	
Previous operation pancreas	Laparoscopic/endoscopic	5	6.3	16	4.2	0.406
	Open	74	93.7	366	95.8	
ASA	I	5	6.3	31	8.1	0.458
	II	41	51.9	219	57.3	
	III/IV	33	41.8	132	34.6	
Defect size	I (< 4 cm)	20	25.3	94	24.6	0.926
	II (4–10 cm)	42	53.2	198	51.8	
	III (> 10 cm)	17	21.5	90	23.6	
EHS classification	Medial	58	73.4	252	66.0	0.438
	Lateral	10	12.7	61	16.0	
	Combined	11	13.9	69	18.1	
Preoperative pain	No	25	31.6	133	34.8	0.583
	Yes	49	62.0	215	56.3	
	Unknown	5	6.3	34	8.9	
Drainage	Yes	11	13.9	278	72.8	< 0.001
	No	68	86.1	104	27.2	
Direct suture	Yes	20	25.3	222	58.1	< 0.001
	No	59	74.7	160	41.9	
Mesh	Yes	77	97.5	344	90.1	0.033
	No	2	2.5	38	9.9	
Risk factors—total	Yes	42	53.2	161	42.1	0.073
	No	37	46.8	221	57.9	
COPD	Yes	6	7.6	25	6.5	0.734
	No	73	92.4	357	93.5	
Diabetes	Yes	20	25.3	83	21.7	0.486
	No	59	74.7	299	78.3	
Aortic aneurysm	Yes	0	0	3	0.8	0.429
	No	79	100	379	99.2	
Immunosuppression	Yes	0	0	7	1.8	0.225
	No	79	100	375	98.2	
Corticoids	Yes	1	1.3	5	1.3	0.975
	No	78	98.7	377	98.7	
Tuxedo	Yes	13	16.5	52	13.6	0.509
	No	66	83.5	330	86.4	
Coagulopathy	Yes	1	1.3	3	0.8	0.675
	No	78	98.7	379	99.2	
Antithrombotic medication	Yes	12	15.2	40	10.5	0.227
	No	67	84.8	342	89.5	
Anticoagulant medication	Yes	3	3.8	5	1.3	0.123
	No	76	96.2	377	98.7	
Liver cirrhosis‡	Yes	0	0	0	0	1.000
	No	2	100	4	100	
Anticoagulants‡	Yes	0	0	0	0	1.000
	No	2	100	4	100	

**Table 5:** Continuous parameters: patient- and procedure-related parameters

		Access route for hernia surgery		*p*
		Minimally invasive	Open	
Age [years]	N/Mean ± SD	79/62.3 ± 11.6	382/61.7 ± 12.8	0.671
BMI [kg/m^2^]	N/Mean ± SD	79/27.2 ± 5.2	382/25.8 ± 4.4	0.031
Duration of operation [min]*	N/Mean [Range of dispersion]	79/84.3 [82.5; 86.0]	374/79.0 [77.2; 80.7]	0.338
Mesh size [cm^2^]*	N/mean [Range of dispersion]	77/340.6 [338.9; 342.3]	343/259.6 [257.3; 262.0]	< 0.001

*Logarithmic transformation: Illustration of the back-transformed mean values and ranges (mean value ± SD)

**Table:**

Parameters by type of access		*N*	NMiss	Mean	SD	Min	Q1	Median	Q3	Max
Age [years]	Laparoscopic surgery	79	0	62.3	11.63	35.0	53.0	61.0	73.0	86.0
	Open surgery	382	0	61.7	12.81	22.0	53.0	63.0	72.0	86.0
	Total	461	0	61.8	12.61	22.0	53.0	63.0	72.0	86.0
BMI [kg/m^2^]	Laparoscopic surgery	79	0	27.2	5.25	17.3	24.3	26.4	28.7	49.0
	Open surgery	382	0	25.8	4.45	15.4	22.8	25.3	28.2	45.4
	Total	461	0	26.1	4.62	15.4	23.0	25.6	28.3	49.0
Duration of operation [min]	Laparoscopic surgery	79	0	96.7	51.58	23.0	58.0	86.0	126.0	263.0
	Open surgery	374	8	92.1	52.53	20.0	56.0	78.0	120.0	380.0
	Total	453	8	92.9	52.34	20.0	58.0	80.0	120.0	380.0
Mesh size [cm^2^]	Laparoscopic surgery	77	2	395.5	239.41	81.0	225.0	300.0	500.0	1500.0
	Open surgery	343	39	348.2	251.16	16.0	150.0	300.0	450.0	1500.0
	Total	420	41	356.9	249.44	16.0	150.0	300.0	500.0	1500.0

**Table 6:** Categorical parameters: postoperative complications and 1-year follow-up outcomes

		Access route for hernia surgery				*p*
		Minimally invasive		Open		
		*n*	%	*n*	%	
Intraoperative complications—total	Yes	3	3.8	5	1.3	0.123
	No	76	96.2	377	98.7	
General complications—total	Yes	0	0	8	2.1	0.194
	No	79	100	374	97.9	
Postoperative complications—total	Yes	4	5.1	36	9.4	0.210
	No	75	94.9	346	90.6	
Complication-related reoperations	Yes	3	3.8	14	3.7	0.955
	No	76	96.2	368	96.3	
Recurrence at 1-year follow-up	Yes	3	3.8	22	5.8	0.483
	No	76	96.2	360	94.2	
Pain on exertion at 1-year follow-up	Yes	17	21.5	80	20.9	0.909
	No	62	78.5	302	79.1	
Pain at rest at 1-year follow-up	Yes	8	10.1	44	11.5	0.722
	No	71	89.9	338	88.5	
Pain requiring treatment at 1-year follow-up	Yes	6	7.6	34	8.9	0.707
	No	73	92.4	348	91.1	
Trocar hernia at 1-year follow-up	Yes	0	0	1	0.3	0.649
	No	79	100	381	99.7	
Secondary hemorrhage at 1-year follow-up	Yes	1	1.3	4	1.0	0.864
	No	78	98.7	378	99.0	
Seroma at 1-year follow-up	Yes	1	1.3	12	3.1	0.359
	No	78	98.7	370	96.9	
Infection at 1-year follow-up	Yes	0	0	7	1.8	0.225
	No	79	100	375	98.2	

**Table 7:** Categorical parameters: intraoperative complications

		Access route for hernia surgery			
		Minimally invasive		Open	
		*n*	%	*n*	%
Bleeding	Yes	1	1.3	1	0.3
	No	78	98.7	381	99.7
Organ injuries	Yes	2	2.5	4	1.0
	No	77	97.5	378	99.0
Vascular	Yes	0	0	0	0
	No	79	100	382	100
Bowel	Yes	1	1.3	4	1.0
	No	78	98.7	378	99.0
Bladder	Yes	0	0	0	0
	No	79	100	382	100
Stomach	Yes	0	0	0	0
	No	79	100	382	100
Spleen	Yes	1	1.3	0	0
	No	78	98.7	382	100
Liver	Yes	0	0	0	0
	No	79	100	382	100
Others	Yes	0	0	0	0
	No	79	100	382	100

**Table 8:** Categorical parameters: general complications

		Access route for hernia surgery			
		Minimally invasive		Open	
		*n*	%	*n*	%
Fever	Yes	0	0	0	0
	No	79	100	382	100
Urinary tract infection	Yes	0	0	0	0
	No	79	100	382	100
Diarrhea	Yes	0	0	2	0.5
	No	79	100	380	99.5
Gastritis	Yes	0	0	0	0
	No	79	100	382	100
Thrombosis	Yes	0	0	0	0
	No	79	100	382	100
Pulmonary embolism	Yes	0	0	0	0
	No	79	100	382	100
Pleural effusion	Yes	0	0	0	0
	No	79	100	382	100
Pneumonia	Yes	0	0	0	0
	No	79	100	382	100
COPD	Yes	0	0	0	0
	No	79	100	382	100
Cardiac insufficiency	Yes	0	0	0	0
	No	79	100	382	100
Coronary heart disease	Yes	0	0	0	0
	No	79	100	382	100
Myocardial infarction	Yes	0	0	0	0
	No	79	100	382	100
Renal insufficiency	Yes	0	0	0	0
	No	79	100	382	100
Hypertensive crisis	Yes	0	0	1	0.3
	No	79	100	381	99.7
Patient deceased	Yes	0	0	0	0
	No	79	100	382	100
Others	Yes	0	0	5	1.3
	No	79	100	377	98.7

**Table 9:** Categorical parameters: postoperative complications

		Access route for hernia surgery			
		Minimally invasive		Open	
		*n*	%	*n*	%
Bleeding	Yes	1	1.3	13	3.4
	No	78	98.7	369	96.6
Seroma	Yes	0	0	13	3.4
	No	79	100	369	96.6
Prolonged ileus or obstruction	Yes	1	1.3	0	0
	No	78	98.7	382	100
Bowel injury/anastomotic insufficiency	Yes	0	0	2	0.5
	No	79	100	380	99.5
Wound healing disorder	Yes	0	0	10	2.6
	No	79	100	372	97.4
Infection	Yes	2	2.5	3	0.8
	No	77	97.5	379	99.2
